# Chemical Composition and Fatty Acid Profile of the *Pectoralis major* Muscle in Broiler Chickens Fed Diets with Full-Fat Black Soldier Fly (*Hermetia illucens*) Larvae Meal

**DOI:** 10.3390/ani12040464

**Published:** 2022-02-14

**Authors:** Tomasz Daszkiewicz, Daria Murawska, Dorota Kubiak, Jolanta Han

**Affiliations:** 1 Department of Commodity Science and Processing of Animal Raw Materials, University of Warmia and Mazury in Olsztyn, Oczapowskiego 5, 10-719 Olsztyn, Poland; dorota.kubiak@uwm.edu.pl (D.K.); jolantahan8@gmail.com (J.H.); 2 Department of Commodity Science and Animal Improvement, University of Warmia and Mazury in Olsztyn, Oczapowskiego 5, 10-719 Olsztyn, Poland; daria.murawska@uwm.edu.pl

**Keywords:** insect meal, animal feed, chickens, meat quality

## Abstract

**Simple Summary:**

Insect meal (processed insect protein) can effectively cater to the growing demand for protein sources in animal diets. However, insect meals have to be thoroughly analyzed before they can be incorporated into the diets of monogastric animals (such as poultry) because animal nutrition is directly correlated with the quality (including chemical composition) of meat, which was confirmed by the present study. Further research is needed to determine the efficacy of full-fat *Hermetia illucens* (*HI*) larvae meal in broiler chicken nutrition. Lower dietary inclusion levels of insect meal than those analyzed in this study, and insect meal with a modified fatty acid profile should be investigated to optimize the nutritional value and health benefits of poultry meat.

**Abstract:**

The aim of this study was to determine the effect of full-fat *Hermetia illucens* (*HI*) larvae meal used as a substitute for 50%, 75% and 100% of soybean meal (SBM) in diets fed to male Ross 308 broiler chickens on the proximate chemical composition and fatty acid profile of the *Pectoralis major* (*PM*) muscle. The muscles of chickens fed *HI* larvae meal were characterized by a higher (*p* < 0.05) total concentration of pigments and lower (*p* < 0.05) ash content. At the lowest dietary inclusion rate (50%) of *HI* larvae meal, the *PM* muscle had a lower (*p* < 0.05) content of fat and collagen, compared with the remaining treatments. An analysis of the fatty acid profile of the *PM* muscle revealed that the total concentrations of saturated fatty acids increased (*p* < 0.05) and the total concentrations of polyunsaturated fatty acids decreased (*p* < 0.05) with increasing inclusion levels of *HI* larvae meal. The changes in the fatty acid profile of muscles in birds receiving *HI* larvae meal led to a decrease in the nutritional value of fat. The present findings indicate that the ≥50% inclusion rate of full-fat *HI* larvae meal as a protein source alternative to SBM in broiler chicken diets is too high due to its negative effect on the fatty acid profile of meat.

## 1. Introduction

Rapid population growth increases the demand for food. The dynamic economic growth of many countries has induced changes on the global food market and has contributed to a higher demand for animal-based products [1]. Poultry production is one of the most rapidly developing sectors of the livestock industry. According to the Food and Agriculture Organization of the United Nations [2], global poultry production reached 131.6 million tons in 2019 and was expected to increase to 133.3 million tons in 2020.

The dynamic growth of poultry farming has also increased the demand for high-protein feed components. At present, soybean meal (SBM) is the main source of protein in intensive poultry production systems [3]. According to Stein et al. [4], SBM is widely used in animal diets on account of its high protein content and a unique amino acid profile that complements the amino acid profile of cereal grains. Soybean meal for animal diets is produced mainly from genetically modified (GM) soybean plants. In 2018, GM soybeans had a 78% share of the world production of GM crops [5]. The area under soybean cultivation varies across continents due to the climate and soil requirements of soybean plants as well as the regulations and restrictions on GM crops [6]. Brazil, the USA and Argentina are the world’s leading producers of soybeans [7].

The concerns over future SBM supplies, accompanied by growing demand and prices, have spurred the search for alternative protein sources in animal diets. Numerous attempts have been made to substitute soybeans with other plant seeds, including oilseed crops such as canola and sunflower, cotton seeds, peanuts, and legume seeds such as field beans, peas and lupins. However, these crops are characterized by lower levels of digestible energy, a lower protein content and a less favorable amino acid profile than SBM [4]. The seeds of some crops contain antinutritional factors, which limits their use for feed production [8]. New processing technologies are being developed to utilize other sources of feed proteins. Insect meal appears to be a highly promising alternative protein source in animal nutrition.

The growing popularity of edible insects as an alternative ingredient of livestock rations can be attributed to their high content of protein, essential amino acids, fat, minerals and vitamins, as well as the fact that insect farming exerts a smaller impact on the environment than the production of other feed protein sources [9,10]. The influence of different dietary inclusion levels of insect meal and fat on the performance of various livestock species and meat quality has been widely investigated [11]. Published data indicate that insect protein could be used as a substitute for vegetable protein (mainly SBM) in animal diets. However, the optimal addition of insect meal to animal diets for high-quality meat remains to be determined.

*Hermetia illucens (HI)*, *Tenebrio molitor* and *Musca domestica* are the most promising insect species for the industrial production of poultry feeds [10,12]. *Hermetia illucens* has been most extensively researched as a source of dietary protein. However, further research is needed because the chemical composition of diets fed to monogastric animals affects the chemical composition of meat, including its fatty acid profile. The research hypothesis tested in this study postulates that *HI* larvae meal can significantly influence the nutritional value and health benefits of meat (associated with a more favorable fatty acid profile). The aim of the present study was to determine the effect of full-fat *HI* larvae meal used as a substitute for 50%, 75% and 100% of SBM protein in broiler chicken diets on their growth performance, and on the proximate chemical composition and fatty acid profile of meat.

## 2. Materials and Methods

### 2.1. Birds, Management and Diets

Pursuant to the provisions of the Polish law and Directive 2010/63/EU [13], the experiment did not require the approval of the Local Ethics Committee for Animal Experimentation (Decision of the Ethics Committee: Ethic Committee Name: Local Ethics Committee, Approval Code: LKE.065.22.2019, Approval Date: 29 April 2019). The experiment was performed on 384 male Ross 308 broiler chickens. Throughout the experiment (1 to 42 days of age), the birds were housed in an experimental facility at the Faculty of Animal Bioengineering, University of Warmia and Mazury in Olsztyn (Poland), equipped with mechanical ventilation and artificial lighting (a photoperiod of 6 h dark and 18 h light). Infrared heat lamps were used during the first three weeks to maintain the optimal temperature, according to the best practice recommendations [14]. The chickens were vaccinated against Newcastle disease, Marek’s disease, avian infectious bronchitis and coccidiosis.

The chickens were transported to the experimental facility and placed in pens measuring 1.10 × 1.25 m. The pens were bedded with straw pellets and equipped with automatic drinkers and feeders. The birds were randomly divided into four groups-dietary treatments (8 pens/treatment and 12 birds/pen) in order to determine the effect of processed animal protein (PAP) from full-fat *Hermetia illucens* larvae meal (PAP-*HI*) (HiProMine S.A., Robakowo, Poland) on meat quality. Control group chickens were fed diets without PAP-*HI* (0 PAP-*HI*), whereas birds in three experimental groups received diets where SBM protein was replaced with PAP-*HI* in the amount of 50% (50% PAP-*HI*), 75% (75% PAP-*HI*) and 100% (100% PAP-*HI*). The ingredients, chemical composition and energy value of diets fed to broiler chickens are presented in our previous paper [15]. The fatty acid profile of *HI* larvae meal is shown in Table 1, whereas the fatty acid profile of diets is presented in Table 2. Feed and water were available ad libitum.

### 2.2. Sample Collection

Broiler chickens (2 birds/pen, 16 birds/treatment, 64 birds in total) were slaughtered at 42 days of age (stunning with the electrical water-bath stunner with 0.12 mA per bird applied for minimum 4 s and bleeding by severing the jugular vein). Their growth performance and selection for slaughter are presented in our previous paper [15]. After post-slaughter processing, the carcasses were chilled at 4 °C for around 24 h. Chilled carcasses were dissected, and the *Pectoralis major* (*PM*) muscles were cut out. Eight muscles of control group chickens and eight muscles of birds from each experimental group (1 bird/pen, 8 birds/treatment, 32 birds in total) were packaged in string polyethylene bags and transported to the laboratory. In the laboratory, left *PM* muscles were analyzed for the chemical composition and the fatty acid profile of intramuscular fat (IMF). Until analysis (48 h postmortem), the muscles packaged in string polyethylene bags were stored in a cold storage chamber (dark, 4 °C). Before the chemical analysis, muscle samples were passed through a 3 mm plate in a meat grinder (Tefal NE 109838/J90-0320, Groupe SEB, Lourdes, France), and were thoroughly mixed with a hand immersion blender (Tefal HB665, Groupe SEB, Mayenne, France).

### 2.3. Laboratory Analysis

#### 2.3.1. Proximate Chemical Composition

The proximate chemical composition of meat samples was determined according to the procedure of AOAC [16]. Water content was determined by sample drying at a temperature of 105 °C to a constant weight. Crude protein content was determined by the Kjeldahl method (Kjeltec™ 2200 Auto Distillation Unit, FOSS Analytical, Hilleroed, Denmark). The conversion factor of 6.25 was used to convert N values to crude protein content. Crude fat content was determined by the Soxhlet method (Soxtec™ 2050 Auto Fat Extraction System, FOSS Analytical, Hilleroed, Denmark). Extraction was performed with diethyl ether as the solvent. Ash content was determined by incineration to a constant weight at 550 °C in a muffle furnace.

#### 2.3.2. Collagen Content

The collagen content of meat was determined based on the hydroxyproline content [17], which was converted into total collagen content using a conversion factor of 7.25 [18].

#### 2.3.3. Total Content of Muscle Pigments

The total content of muscle pigments was determined as described by Hrynets et al. [19]. Meat samples weighing 10 g were combined with 40 mL acetone, 1 mL HCl and 1 mL distilled H_2_O, and were mixed (IKA^®^ Werke GmbH & Co. KG, Staufen, Germany) in a sealed plastic test tube for 3 min. The homogenized samples were left at room temperature for 1 h, and then filtered through filter paper (Whatman No.1). Absorbance was measured in the filtrate (Specord^®^ 40 spectrophotometer, Analytik Jena AG, Jena, Germany) at a wavelength of 640 nm, against the acid-acetone blank. The absorbance value was multiplied by 17.18. The total content of heme pigments was expressed in milligrams of pigments per gram of meat.

#### 2.3.4. Fatty Acid Profile

The fatty acid profile was determined in IMF extracted by the Soxhlet method. The methylation of fatty acids was carried out as described by Żegarska et al. [20]. Fat samples were transferred into glass ampoules with the addition of 1.5 mL of a chloroform, methanol and sulfuric acid mixture (100:100:1 *v*/*v*). The ampoules were sealed in the flame of a gas burner and placed in a thermostat (105 °C) for 2 h. Fatty acids were separated on the VARIAN CP-3800 gas chromatograph (Varian Inc., Palo Alto, CA, USA) coupled to a flame ionization detector (FID), with a capillary column (length—50 m, inner diameter—0.25 mm, liquid phase—CP-Sil 88, film thickness—0.25 μm). The column temperature was 200 °C. Injector and detector temperatures were 225 and 250 °C, respectively. The carrier gas was helium (flow rate—1.2 mL/min). Fatty acids were identified by comparing the retention times of methyl easters in the analyzed samples and the standard mixture of fatty acid methyl esters (Sigma, St. Louis, MO, USA). The relative content of fatty acids was determined based on internal normalization.

### 2.4. Statistical Analysis

The results were analyzed statistically using the STATISTICA program ver. 13.3 (TIBCO Software Inc., Palo Alto, CA, USA). One-way ANOVA was performed to determine the effects of the experimental factor (diet with different levels of insect protein). The significance of differences between group means was estimated by Tukey’s test at a confidence level of *p* < 0.05. In addition, polynomial contrasts were used to further determine linear and quadratic responses to increasing levels of full-fat insect meal (*Hermetia illucens*) in the diet. Results were considered significant if the *p*-values were less than 0.05.

## 3. Results

### 3.1. Proximate Chemical Composition

In all groups, samples of the *PM* muscle had high water contents (Table 3), at 74.86% on average. Water content was highest in the muscles of broilers fed diets containing 50% PAP-*HI*, and lowest in the muscles of birds receiving 75% PAP-*HI*. The difference between the means of these groups was significant (*p* < 0.05).

The high total concentration of dry matter components in the *PM* muscle resulted from its high protein content (22.47% on average) (Table 3). The protein content of meat showed quadratically increased (*p* = 0.023), with a maximum observed for the 75% PAP-*HI* group.

The fat content in the *PM* muscle (Table 3) was characterized by a quadratic response (*p* = 0.047) to increasing PAP-*HI* in the diet. In the muscles of birds fed the diet with the lowest inclusion rate (50%) of PAP-*HI*, fat content was significantly (*p* < 0.05) lower than in the control group. The content of this component in the muscle of broiler chickens in the experimental groups fed diets containing 75% and 100% PAP-*HI* was slightly lower (*p* > 0.05).

The inclusion of PAP-*HI* in the broiler chicken diet affected (linear response, *p* < 0.001) the concentrations of ash (minerals) in the *PM* muscle (Table 3). The content of this component in meat was considerably lower (*p* < 0.05) in the experimental groups than in the control group.

### 3.2. Collagen Content

In the present study, the collagen content of the *PM* muscle was lowest in broilers fed 50% PAP-*HI* (Table 3). The mean value of this group was significantly (*p* < 0.05) lower compared with the value of the control group and the experimental group receiving 75% PAP-HI.

### 3.3. Total Content of Muscle Pigments

Total concentration of heme pigments in the muscles of broiler chickens showed a linear and quadratic increase response (*p* < 0.001 and *p =* 0.008, respectively) to increasing PAP-*HI* levels, with a maximum for the diet with 75% PAP-*HI* (Table 3). The pigments’ content in the *PM* muscles of broiler chickens in the control group was lower (*p* < 0.05) compared with the muscles of birds fed diets containing 75% and 100% PAP-*HI*. A significant (*p* < 0.05) difference in the concentration of muscle pigments was also found between broilers receiving diets with 50% and 75% PAP-*HI*.

### 3.4. Fatty Acid Profile

An analysis of the fatty acid profile of IMF in broilers fed diets containing PAP-*HI* revealed a significantly (*p* < 0.05) higher proportion of saturated fatty acids (SFAs), compared with the control group (Table 4). Their total content increased (linear and quadratic response, *p* < 0.001) with increasing dietary inclusion levels of PAP-*HI*. This relationship was observed for fatty acids with shorter carbon chains, i.e., lauric acid (C12:0) and myristic acid (C14:0). Linear and quadratic responses (*p* < 0.001) were also observed for pentadecanoic acid (C15:0), with a maximum for the chickens fed a diet containing 75% PAP-*HI*, and a quadratic response (*p* < 0.001) for margaric acid (C17:0), with a maximum for the diets with the inclusion of 50% and 75% PAP-*HI*. However, the differences between mean values were very small, and their significance could result from a very low variation in the amounts of C15:0 and C17:0. Among SFAs with longer carbon chains, a decreased (linear and quadratic response, *p* < 0.001) concentration of stearic acid (C18:0) was noted with increasing PAP-*HI* in the diet. A linear decrease (*p =* 0.035) was observed for arachidic acid (C20:0) also.

An analysis of monounsaturated fatty acids (MUFAs) (Table 5) revealed an increase in concentrations of myristoleic acid (C14:1) (linear and quadratic response, *p* < 0.001) and palmitoleic acid (C16:1) (quadratic response, *p* < 0.001) in the IMF of broilers with increasing PAP-*HI* in the diets. A linear increase (*p* = 0.040) was shown for heptadecenoic acid (C17:1). The concentrations of MUFAs with longer carbon chains, i.e., oleic acid (C18:1) and gadoleic acid (C20:1) decreased for increasing PAP-*HI* levels in the diets. Linear and quadratic responses (*p* < 0.001 and *p* = 0.006, respectively) were observed for C18:1, and a linear response (*p* < 0.001) for C20:1. No significant (*p* > 0.05) differences in the proportion of MUFAs in the IMF of chickens were found between group means.

The proportion of polyunsaturated fatty acids (PUFAs) was significantly (*p* < 0.05) lower in the IMF of chickens from the experimental groups than in the IMF of control group birds (Table 5). It was also negatively correlated with the dietary inclusion levels of PAP-*HI* (linear and quadratic effect, *p* < 0.001). However, the above relationship was not observed in all analyzed PUFAs. An increase in the inclusion rate of PAP-*HI* was accompanied by a decrease (linear and quadratic response, *p* < 0.001) in the concentrations of linoleic acid (C18:2), linolenic acid (C18:3) and, to a lesser degree (linear response, *p* = 0.005), C20:2. It should also be noted that the decrease in the amounts of C18:2 and C18:3 was statistically not significant (*p* > 0.05) when the PAP-*HI* content of the diet was increased from 50% to 75%. The concentrations of arachidonic acid (C20:4), timnodonic acid (C20:5) and clupanodonic acid (C22:5) did not differ significantly (*p* > 0.05) between groups. The only PUFA whose concentration increased (linear and quadratic effect, *p* = 0.020 and *p* = 0.011, respectively) in response to the incorporation of PAP-*HI* into the diet was docosahexaenoic acid (C22:6). The increase in the concentration of this acid was significant (*p* < 0.05), relative to the control group, in the experimental groups receiving diets with 50% and 75% PAP-*HI*.

The differences in the fatty acid profile of the IMF in broiler chickens led to differences in the parameters characterizing the nutritional value of fat (Table 6). The addition of PAP-*HI* to broiler diets had a negative effect on the nutritional value of fat (linear and quadratic response, *p* < 0.001). The IMF of birds in the experimental groups was characterized by significantly (*p* < 0.05) lower values of the ratios between unsaturated and saturated fatty acids (UFA/SFA), MUFA/SFA, PUFA/SFA, and between hypocholesterolemic and hypercholesterolemic fatty acids (DFA/OFA), and it had a lower proportion of essential fatty acids (EFAs) and index of nutritional value. We did not find significant (*p* > 0.05) differences between the average values of the MUFA/SFA ratios calculated for the experimental groups. In addition, no significant (*p* > 0.05) differences were found between the average values of the UFA/SFA and PUFA/SFA ratios, as well as between mean values of EFAs in the IMF of broilers receiving diets with 50% and 75% PAP-*HI*. Among the experimental groups, the nutritional value index was significantly (*p* < 0.05) higher in the IMF of chickens fed the diet with 50% PAP-*HI*, compared with the IMF of birds fed the diet containing 100% PAP-*HI*.

## 4. Discussion

### 4.1. Proximate Chemical Composition

The results of an analysis of the water and protein content of the *PM* muscle in broiler chickens were similar to those reported by Altmann et al. [21] (73.88–74.29% and 20.52–21.07%, respectively) and Schiavone et al. [22] (75.24–76.14% and 22.28–23.09%, respectively). The fat content of breast muscles in broiler chickens noted by Schiavone et al. [22] (1.56–1.75%) was also comparable with that determined in the current experiment. A considerably higher fat content of broiler chicken meat (3.40–3.41%) was reported by Altmann et al. [21]. The ash content of breast muscles (1.23–1.33%) determined by Schiavone et al. [22] was higher than that observed in the present study. It should be stressed that the results of an analysis of the chemical composition of the *PM* muscle cannot be directly compared with the findings of other authors due to the effects exerted by various factors such as broiler chicken nutrition. Such comparisons can only provide approximate data, whereas the effects of the experimental factor (the inclusion of *HI* larvae meal in broiler chicken diets) on the analyzed parameters can be directly compared. *Hermetia illucens* larvae meal had no significant influence on the proximate chemical composition of raw broiler meat [21] or cooked broiler meat [23]. Schiavone et al. [22] found that the water content of breast muscles decreased linearly, and the protein content increased linearly with increasing inclusion levels of *HI* larvae meal in broiler chicken diets, whereas the concentrations of fat and ash did not change significantly. Other studies investigating different animal species fed *HI* larvae meal ([24]—pacific white shrimp, [25]—broiler quails, [26]—rainbow trout, [27]—Barbary partridges) revealed no significant differences in the proximate chemical composition of meat, relative to control group animals fed conventional diets. Similar conclusions were formulated in experiments where *HI* larvae fat was used as a substitute for soybean oil in broiler chicken diets [28,29,30]. In the present study, an analysis of the chemical composition of broiler chicken meat revealed that *HI* larvae meal exerted an unambiguous effect only on ash content. The concentration of ash in the *PM* muscle decreased (linear response) with increasing inclusion rates of *HI* larvae meal, most likely because full-fat meal was used in this experiment. According to Cullere et al. [25], defatting increases mineral concentrations in insect meal, which in turn may increase the ash content of meat. However, such a relationship was not observed by Popova et al. [31] who found no significant differences in the ash content of meat from broiler chickens fed diets containing partially defatted and full-fat *HI* larvae meal.

### 4.2. Collagen Content

Collagen affects the organoleptic (tenderness) [32] and functional (water binding during heat treatment) properties of meat [33]. Therefore, collagen content is often determined in studies investigating meat quality, particularly in ruminants (cattle, sheep). Listrat et al. [34] demonstrated that collagen represents 0.75% to 2.0% of muscle dry weight in poultry. According to the cited authors, the factors affecting the collagen content of poultry meat remain insufficiently researched because birds are slaughtered at a relatively early stage of physiological development when intramuscular collagen is not significantly cross-linked. The information about the effect of the use of insect meal (processed insect protein) on collagen content in the meat of broiler chickens is lacking too. As a result, it is difficult to compare the current findings with the results of other studies analyzing the influence of insect meal on the meat quality of broiler chickens, and to explain the observed decrease in collagen concentration in the muscles of birds fed diets containing 50% PAP-*HI*. Further research is needed to verify the above observations.

### 4.3. Total Content of Muscle Pigments

Color is one of the key attributes of meat, affecting consumers’ purchase decisions [35]. Meat color is determined by the content and chemical form of myoglobin, as well as the content of hemoglobin and cytochrome C. In the present study, the total concentration of heme pigments in the *PM* muscle of broiler chickens was consistent with the values cited in the literature [36,37]. The present findings cannot be compared with the results of other studies investigating *HI* larvae meal because they did not include the determination of muscle pigments. In the current experiment, a linear and quadratic increase was observed for the total concentration of heme pigments in the muscles of chickens fed diets with insect meal. According to Dierenfeld and King [38], *HI* larvae contain higher concentrations of minerals, including Fe, than other edible insects used as animal feed. A higher supply of Fe, which is present in the heme group of heme pigments, may be linked with the concentration of heme pigments in meat. However, it should be noted that the content of minerals in *HI* larvae (and, in consequence, in *HI* larvae meal) varies depending on the type of substrate (food) on which they are reared and their age [39].

### 4.4. Fatty Acid Profile

In a review summarizing the results of published research on the nutritional value of *HI* larvae as animal feed, Barragan-Fonseca et al. [39] demonstrated that *HI* larvae and prepupae contain 58–72% of SFAs and 19–40% of MUFAs and PUFAs in the total fatty acid pool, which directly translates into their concentrations in *HI* larvae meal. The cited authors also found that the fatty acid profile of larvae and prepupae could be affected by the fatty acid composition of their diet. It should be noted that *HI* larvae prefer foods with a high fat content to build up body fat reserves necessary to complete development [40]. The fatty acid profile determined in the present study (Table 4 and Table 5) could be expected since SFAs predominate in *HI* larvae meal (Table 1, [39]) and broiler chickens are monogastric animals whose diets affect meat composition. Our results corroborate the findings of Cullere et al. [25] and Popova et al. [31], who analyzed the breast muscles of Japanese quails and broiler chickens, respectively, and found that the proportion of SFAs was higher in the muscles of birds fed *HI* larvae meal. In contrast, Schiavone et al. [22] did not observe a significant increase (not a linear and quadratic response) in SFAs in the breast muscles of chickens receiving *HI* larvae meal. In the present study, lauric acid (C12:0) and myristic acid (C14:0) were the SFAs whose content of the *PM* muscle of broiler chickens increased (a linear and quadratic response) considerably with increasing dietary inclusion levels of *HI* protein, which corroborates the findings of Schiavone et al. [22] and Cullere et al. [25]. An increase in the proportion of the above acids, relative to their amounts in meat from broilers fed conventional diets, was also reported by Popova et al. [31]. In all of the cited studies, an increase was noted in the concentration of palmitic acid (C16:0); however, this was significant only in the meat of broiler quails analyzed by Cullere et al. [25]. An increase in the concentrations of the above fatty acids is undesirable because they exert atherogenic effects [41] by inhibiting the expression of the LDL (low-density lipoprotein) receptor gene, thus increasing LDL cholesterol synthesis and total cholesterol levels [42].

Schiavone et al. [22] found that the dietary inclusion rate of *HI* larvae meal was inversely proportional (a linear effect) to the concentration of stearic acid (C18:0) in the breast muscles of broiler chickens, which was also observed in the current experiment. Cullere et al. [25] also noted such a relationship, but it was less pronounced. In contrast, Popowa et al. [31] found no significant changes in the concentration of C18:0 in the meat of broilers fed diets containing *HI* larvae meal.

In the present study, *HI* larvae meal had no significant effect on the proportion of MUFAs in the *PM* muscle of broiler chickens, which is consistent with the findings of Popova et al. [31] who also analyzed the breast muscles of broilers. However, the cited authors demonstrated that the proportion of MUFAs increased in the thigh muscles of birds fed *HI* larvae meal, compared with the control group. An increase in the proportion of MUFAs in the breast muscles of broiler chickens and broiler quails receiving defatted *HI* larvae meal was reported by Schiavone et al. [22] and Cullere et al. [25], respectively. All of the cited studies revealed that the dietary supplementation with *HI* larvae meal was inversely proportional to the total concentration of PUFAs in broiler chicken meat, which was also noted in the current experiment.

The quality of meat from monogastric animals, including the fatty acid profile, should be evaluated in view of their nutritional status. In studies investigating insect protein sources, including the present study, the dietary inclusion rates of full-fat or defatted insect meal are important considerations. Popova et al. [31] demonstrated that the proportion of SFAs tended to be higher in the meat of broilers fed full-fat *HI* larvae meal than in birds receiving partially defatted meal. The effect of *HI* larvae fat on the fatty acid profile of broiler chicken meat, including a considerably higher proportion of SFAs and a lower proportion of PUFAs, was noted by Schiavone et al. [28], Cullere et al. [29] and Kim et al. [30]. However, the results of analyses of the proportion of MUFAs are inconclusive—the cited studies revealed no significant changes as well as a decrease and an increase in the total concentration of MUFAs in meat, respectively.

The fatty acid profile of meat is the outcome of absorption, de novo synthesis and β-oxidation of fatty acids [43]. Poureslami et al. [44] found that the de novo synthesis of SFAs and MUFAs was negatively correlated (R^2^ = 0.56) with the dietary intake of the above fatty acids in broiler chickens. According to the cited authors, high dietary inclusion levels of SFAs and MUFAs may reduce the biosynthesis of selected MUFAs, in particular 18:1 n-9, due to the inhibition of elongase activity. They also demonstrated that an increased dietary supply of SFAs and MUFAs was positively correlated (R^2^ = 0.97) with their β-oxidation. The above findings may explain the results of the present study where the proportion of MUFAs with longer carbon chains (C18:1 and C20:1) decreased but the proportion of medium-chain fatty acids (C14:1 and C16:1) increased in the *PM* muscle of broiler chickens fed diets containing PAP-*HI*.

The undesirable changes in the fatty acid profile of the *PM* muscle of broiler chickens fed *HI* larvae meal, noted in this study, were accompanied by a deterioration in the nutritional value of IMF. The most popular indicator of the nutritional value of food products is the PUFA/SFA ratio [41]. Its value in the meat of control group birds (1.40) did not differ from those reported for the breast muscles of Ross 308 broilers by other authors ([22]—1.46, [28]—1.14, [30]—1.08, [31]—1.25). However, the decrease in the PUFA/SFA ratio in the IMF of birds fed *HI* larvae meal, noted in the present study, was much greater than the values reported by Schiavone et al. [22] and Popova et al. [31]. However, it should be stressed that in the cited studies, the dietary inclusion levels of insect meal were considerably lower (5%, 10% and 15% of defatted meal, and 5% of partially defatted and full-fat meal, respectively) than in the current experiment. Nevertheless, Popova et al. [31] reported that the addition of 5% of partially defatted and full-fat *HI* larvae meal decreased the PUFA/SFA ratio in the IMF of breast muscles to 0.98 and 0.99, respectively. In turn, Schiavone et al. [22] demonstrated that the highest inclusion rate (15%) of defatted *HI* larvae meal decreased the PUFA/SFA ratio to 1.23. The influence of defatting *HI* larvae meal on the fatty acid profile of IMF was investigated in studies where broiler chickens were fed diets with different levels of *HI* larvae fat used as a substitute for soybean oil [28,29,30]. They revealed that the PUFA/SFA ratio in meat decreased substantially with increasing dietary inclusion rates of insect fat. Rubayet Bostami et al. [45] demonstrated that the addition of animal fat (chicken fat, beef fat/tallow, beef fat/tallow and pork fat/lard, pork fat/lard) to broiler diets leads to a decrease in the PUFA/SFA ratio in meat.

According to Chen and Liu [41], the DFA/OFA ratio could be a more reliable indicator for assessing the impact of the fatty acid profile of foods on the incidence of cardiovascular disease than the PUFA/SFA ratio. In the current study, full-fat *HI* larvae meal used as a substitute for 50% and 100% of SBM in broiler chicken diets caused a nearly twofold decrease and an over 2.5-fold decrease in the DFA/OFA ratio in the IMF of the *PM* muscle, respectively, relative to the control treatment. A clearly negative effect of a 5% inclusion rate of partially defatted and full-fat *HI* larvae meal to broiler chicken diets on the DFA/OFA ratio was also reported by Popova et al. [31]. Interestingly, the observed decrease in the value of this indicator was not affected by the fat content of the analyzed meal. According to Schiavone et al. [22], modification of the rearing substrate can play an important role in alleviating the potential adverse effect of *HI* larvae meal fed to broiler chickens on the fatty acid profile of their meat.

## 5. Conclusions

The results of this study indicate that the inclusion of full-fat *Hermetia illucens* (*HI*) larvae meal in broiler chicken diets significantly affected the chemical composition of the *Pectoralis major* muscle. *Hermetia illucens* larvae meal used as a substitute for 50%, 75% and 100% of soybean meal exerted negative effects on the nutritional value of intramuscular fat first of all. Therefore, further research is needed to establish the optimal inclusion rates of *HI* larvae meal in broiler chicken diets, lower than those analyzed in this study, in order to produce high-quality meat.

## Figures and Tables

**Table 1:** The fatty acid profile (% of individual fatty acids in the total fatty acid pool) in full-fat *Hermetia illucens* larvae meal.

Item	Content
C10:0	0.86
C12:0	45.97
C14:0	8.70
C15:0	0.15
C16:0	12.21
C16:1	1.91
C17:0	0.20
C17:1	0.20
C18:0	2.53
C18:1 *c9*	11.24
C18:2	14.07
C18:3	1.65
C20:0	0.10
C20:1	0.06
C20:2	0.01
C20:4	0.14
SFAs	70.72
MUFAs	13.41
PUFAs	15.87

SFAs—saturated fatty acids; MUFAs—monounsaturated fatty acids; PUFAs—polyunsaturated fatty acids.

**Table 2:** Proportions (%) of individual fatty acids in the total fatty acid pool in broiler chicken diets.

Item	Diets											
	Starter (1–14 d)				Grower (15–35 d)				Finisher (36–42 d)			
	0% PAP-*HI*	50% PAP-*HI*	75% PAP-*HI*	100% PAP-*HI*	0% PAP-*HI*	50% PAP-*HI*	75% PAP-*HI*	100% PAP-*HI*	0% PAP-*HI*	50% PAP-*HI*	75% PAP-*HI*	100% PAP-*HI*
C10:0	0.02	0.66	0.75	0.78	0	0.36	0.46	0.56	0.02	0.49	0.42	0.42
C12:0	0.38	34.99	38.23	39.62	0.11	20.97	26.70	30.41	0.48	22.68	26.55	28.33
C14:0	0.15	7.14	7.63	8.04	0.12	4.36	5.69	6.48	0.27	4.33	5.49	5.91
C15:0	0.03	0.17	0.17	0.17	0.03	0.10	0.14	0.16	0.33	0.41	0.13	0.11
C16:0	11.72	13.33	13.00	13.12	11.84	11.75	13.32	13.48	25.75	14.38	13.00	13.11
C16:1	0.12	1.67	1.89	1.96	0.11	1.24	1.33	1.50	0.23	1.19	1.39	1.43
C17:0	0.13	0.23	0.23	0.24	0.15	0.19	0.23	0.23	2.80	0.30	0.20	0.18
C17:1	0.07	0.06	0.06	0.06	0.07	0.14	0.05	0.06	0.18	0.06	0.05	0.04
C18:0	4.28	2.62	2.42	2.49	4.34	2.97	3.10	2.86	8.76	2.29	2.98	2.92
C18:1 *c9*	21.55	13.67	13.62	13.30	22.14	17.88	15.62	14.57	36.74	20.67	15.64	14.96
C18:2	54.51	23.01	19.78	18.15	53.89	35.53	29.60	26.42	22.43	29.75	30.20	28.75
C18:3	6.30	2.01	1.87	1.69	6.45	3.90	3.27	2.77	0.66	3.10	3.42	3.19
C20:0	0.41	0.18	0.15	0.15	0.41	0.25	0.23	0.20	0.81	0.46	0.21	0.20
C20:1	0.23	0.14	0.12	0.12	0.22	0.18	0.16	0.15	0.40	0.23	0.16	0.13
C20:2	0.04	0.02	0.02	0.02	0.04	0.03	0.02	0.02	0.10	0	0.02	0.02
C20:4	0.06	0.11	0.03	0.09	0.08	0.16	0.08	0.13	0.02	0.12	0.16	0.30
SFAs	17.12	59.32	62.60	64.62	17.00	40.95	49.87	54.37	39.23	45.34	48.97	51.17
MUFAs	21.97	15.53	15.70	15.43	22.53	19.43	17.15	16.28	37.55	22.15	17.22	16.56
PUFAs	60.91	25.15	21.70	19.95	60.47	39.62	32.97	29.35	23.22	32.97	33.80	32.27

PAP-*HI*—processed animal protein (PAP) in the form of full-fat *Hermetia illucens* (*HI*) larvae meal used as a substitute (0%, 50%, 75%, 100%) for soybean meal (SBM) protein in broiler chicken diets; SFAs—saturated fatty acids; MUFAs—monounsaturated fatty acids; PUFAs—polyunsaturated fatty acids.

**Table 3:** Chemical composition (arithmetic means and SEM) of the breast muscles of broiler chickens (n = 8) fed diets with graded levels of insect protein (*Hermetia illucens*).

Item	Dietary Treatments				SEM	*p*-Value	*p*-Value	
	0% PAP-*HI*	50% PAP-*HI*	75% PAP-*HI*	100% PAP-*HI*			Linear	Quadratic
Water (%)	74.97 ^ab^	75.18 ^b^	74.31 ^a^	75.00 ^ab^	0.110	0.021	0.379	0.241
Protein (%)	22.19	22.46	22.97	22.26	0.110	0.050	0.458	0.023
Fat (%)	2.09 ^a^	1.34 ^b^	1.73 ^ab^	1.66 ^ab^	0.091	0.027	0.228	0.047
Ash (%)	1.15 ^a^	1.12 ^b^	1.12 ^b^	1.10 ^b^	0.005	<0.001	<0.001	0.358
Collagen (%)	0.46 ^a^	0.36 ^b^	0.47 ^a^	0.44 ^ab^	0.013	0.018	0.557	0.146
Muscle pigments (mg/g meat)	0.20 ^c^	0.24 ^bc^	0.29 ^a^	0.26 ^ab^	0.007	<0.001	<0.001	<0.008

PAP-*HI*—processed animal protein (PAP) in the form of full-fat *Hermetia illucens* (*HI*) larvae meal used as a substitute (0%, 50%, 75%, 100%) for soybean meal (SBM) protein in broiler chicken diets. ^a–c^ Values within a row with different superscript lowercase letters are significantly different (*p* < 0.05).

**Table 4:** Proportions (%) of saturated fatty acids in the total fatty acid pool (arithmetic means and SEM) in the intramuscular fat of broiler chickens (n = 8) fed diets with graded levels of insect protein (*Hermetia illucens*).

Item	Dietary Treatments				SEM	*p*-Value	*p*-Value	
	0% PAP-*HI*	50% PAP-*HI*	75% PAP-*HI*	100% PAP-*HI*			Linear	Quadratic
C 12:0	0.07 ^d^	8.74 ^c^	11.74 ^b^	14.20 ^a^	1.229	<0.001	0.00	<0.001
C 14:0	0.49 ^d^	4.07 ^c^	5.03 ^b^	5.49 ^a^	0.452	<0.001	0.00	<0.001
C 15:0	0.09 ^b^	0.15 ^a^	0.16 ^a^	0.14 ^a^	0.007	<0.001	<0.001	<0.001
C 16:0	19.78	20.67	20.18	20.81	0.174	0.127	0.085	0.692
C 17:0	0.20 ^b^	0.23 ^a^	0.23 ^a^	0.19 ^b^	0.005	<0.001	0.581	<0.001
C 18:0	8.41 ^a^	7.81 ^a^	6.72 ^b^	6.24 ^b^	0.226	<0.001	<0.001	0.793
C 20:0	0.16	0.17	0.14	0.12	0.008	0.100	0.035	0.399
C 22:0	0.27	0.26	0.25	0.30	0.011	0.385	0.361	0.161
SFAs	29.46 ^d^	42.09 ^c^	44.43 ^b^	47.51 ^a^	1.589	<0.001	<0.001	<0.001

PAP-*HI*—processed animal protein (PAP) in the form of full-fat *Hermetia illucens* (*HI*) larvae meal used as a substitute (0%, 50%, 75%, 100%) for soybean meal (SBM) protein in broiler chicken diets; SFAs—saturated fatty acids. ^a–d^ Values within a row with different superscript lowercase letters are significantly different (*p* < 0.05).

**Table 5:** Proportions (%) of unsaturated fatty acids in the total fatty acid pool (arithmetic means and SEM) in the intramuscular fat of broiler chickens (n = 8) fed diets with graded levels of insect protein (*Hermetia illucens*).

Item	Dietary Treatments				SEM	*p*-Value	*p*-Value	
	0% PAP-*HI*	50% PAP-*HI*	75% PAP-*HI*	100% PAP-*HI*			Linear	Quadratic
C 14:1	0.06 ^d^	0.44 ^c^	0.58 ^b^	0.71 ^a^	0.056	<0.001	<0.001	<0.001
C 16:1	2.32 ^d^	3.07 ^c^	3.47 ^b^	4.25 ^a^	0.168	<0.001	<0.001	0.913
C 17:1	0.05	0.12	0.14	0.15	0.018	0.160	0.040	0.367
C 18:1	26.80 ^a^	23.42 ^b^	22.45 ^b^	22.68 ^b^	0.478	<0.001	<0.001	0.006
C 18:2	34.92 ^a^	25.41 ^b^	23.96 ^b^	20.29 ^c^	1.256	<0.001	<0.001	<0.001
C 18:3	3.67 ^a^	2.33 ^b^	2.20 ^b^	1.82 ^c^	0.162	<0.001	<0.001	<0.001
C 20:1	0.26 ^a^	0.23 ^ab^	0.20 ^b^	0.19 ^c^	0.008	<0.001	<0.001	0.226
C 20:2	0.41 ^a^	0.37 ^ab^	0.33 ^ab^	0.29 ^b^	0.016	0.038	0.005	0.873
C 20:4	1.60	1.89	1.60	1.51	0.080	0.392	0.459	0.253
C 20:5	0.04	0.03	0.03	0.04	0.003	0.576	0.667	0.196
C 22:5	0.29	0.37	0.34	0.34	0.021	0.558	0.488	0.320
C 22:6	0.12 ^b^	0.24 ^a^	0.25 ^a^	0.22 ^ab^	0.017	0.012	0.020	0.011
MUFAs	29.50	27.27	26.85	27.98	0.380	0.055	0.111	0.021
PUFAs	41.04 ^a^	30.64 ^b^	28.72 ^b^	24.51 ^c^	1.422	<0.001	<0.001	<0.001
UFAs	70.54 ^a^	57.91 ^b^	55.57 ^c^	52.49 ^d^	1.589	<0.001	<0.001	<0.001

PAP-*HI*—processed animal protein (PAP) in the form of full-fat *Hermetia illucens* (*HI*) larvae meal used as a substitute (0%, 50%, 75%, 100%) for soybean meal (SBM) protein in broiler chicken diets; MUFAs—monounsaturated fatty acids; PUFAs—polyunsaturated fatty acids; UFAs—unsaturated fatty acids (UFAs = MUFAs + PUFAs). ^a–d^ Values within a row with different superscript lowercase letters are significantly different (*p* < 0.05).

**Table 6:** Nutritional value index (arithmetic means and SEM) of intramuscular fat in broiler chickens (n = 8) fed diets with graded levels of insect protein (*Hermetia illucens*).

Item	Dietary Treatments				SEM	*p*-Value	*p*-Value	
	0% PAP-*HI*	50% PAP-*HI*	75% PAP-*HI*	100% PAP-*HI*			Linear	Quadratic
UFA/SFA ratio	2.40 ^a^	1.38 ^b^	1.25 ^b^	1.11 ^c^	0.118	<0.001	<0.001	<0.001
MUFA/SFA ratio	1.00 ^a^	0.65 ^b^	0.61 ^b^	0.59 ^b^	0.018	<0.001	<0.001	<0.001
PUFA/SFA ratio	1.40 ^a^	0.73 ^b^	0.65 ^b^	0.52 ^c^	0.079	<0.001	<0.001	<0.001
DFA/OFA ratio	3.76 ^a^	1.92 ^b^	1.65 ^c^	1.42 ^d^	0.213	<0.001	<0.001	<0.001
EFAs	38.59 ^a^	27.74 ^b^	26.16 ^b^	22.11 ^c^	1.417	<0.001	<0.001	<0.001
Nutritional value ^1^	1.78 ^a^	1.51 ^b^	1.45 ^bc^	1.39 ^c^	0.037	<0.001	<0.001	0.001

PAP-*HI*—processed animal protein (PAP) in the form of full-fat *Hermetia illucens* (*HI*) larvae meal used as a substitute (0%, 50%, 75%, 100%) for soybean meal (SBM) protein in broiler chicken diets; UFAs—unsaturated fatty acids (MUFAs + PUFAs); SFAs—saturated fatty acids; MUFAs—monounsaturated fatty acids; PUFAs—polyunsaturated fatty acids; DFAs—hypocholesterolemic fatty acids (UFAs + C18:0); OFAs—hypercholesterolemic fatty acids (SFAs—C18:0); EFAs—essential fatty acids (C18:2 + C18:3); ^1^ (C18:0 + C18:1)/C16:0). ^a–c^ Values within a row with different superscript lowercase letters are significantly different (*p* < 0.05).

## Data Availability

Data available on reasonable request.
